# Supplementary material for: Combining stochastic resetting with Metadynamics to speed-up molecular dynamics simulations
Source: Nat Commun. 2024 Jan 4;15:240. doi: 10.1038/s41467-023-44528-w (PMC10764788; doi:10.1038/s41467-023-44528-w)
Supplement: Supplementary file 1 — Supplementary Information [file 41467_2023_44528_MOESM1_ESM.pdf]

Supplementary Information: Combining stochastic resetting with Metadynamics to speed-up molecular dynamics simulations

Ofir Blumer[1], Shlomi Reuveni[1,2,3], and Barak Hirshberg[1,2,3,*]

[1]School of Chemistry, Tel Aviv University, Tel Aviv 6997801, Israel.
[2]The Center for Computational Molecular and Materials Science, Tel Aviv University, Tel Aviv 6997801, Israel.
[3]The Center for Physics and Chemistry of Living Systems, Tel Aviv University, Tel Aviv 6997801, Israel.
[*]hirshb@tauex.tau.ac.il

Contents

1 Sensitivity to the initial positions 1
2 Estimation of mean first-passage time of transitions between conformers of alanine tetrapeptide 1
3 Transitions between sub-states of alanine tetrapeptide 2
4 Kinetics inference for infrequent MetaD simulations with SR 3
Supplementary References 5

1 Sensitivity to the initial positions

We tested the sensitivity of our results to the initial positions, for the two wells model. In the main text, we present results for simulations initiated at the right minimum, $(x=3, y=0)$ Å. Here, we present results for sets of simulations initiated at different fixed positions, $(x=10,20,30, y=0)$ Å, or with initial positions sampled from the Boltzmann distribution at the right well. Supplementary Fig. 1a presents the initial positions of each set on top of a one-dimensional cross section of the two wells potential along the x-axis. The stars mark the initial x-coordinate, with increasing values from left to right. The shaded area represents the Boltzmann distribution within the right well.

Supplementary Fig. 1b shows the speedup as a function of bias deposition rate for the different sets of Metadynamics (MetaD) simulations. Each set of results matches the appropriate initial positions presented above it in Supplementary Fig. 1a. We first performed standard MetaD simulations, with no stochastic resetting (SR) (green squares). We observe that the speedup from MetaD is similar for all selections of initial positions. We then combined MetaD with SR at the optimal resetting rate (orange triangles), evaluated using Equation 1 of the main text. We obtained the greatest speedups for the initial positions closest to the barrier. For other choices of initial positions, the speedup is similar, and seems to be largely-independent of initial conditions.

2 Estimation of mean first-passage time of transitions between conformers of alanine tetrapeptide

To estimate the mean first-passage time (MFPT) between two conformers of alanine tetrapeptide, we first performed 10^4 unbiased simulations, which were stopped when a transition was observed. However, $\sim 17.5\%$ of them did not show a transition even after $\tau_{max} = 10\,\mu s$. Therefore, unbiased simulations only provided the MFPT for transition times $\tau < \tau_{max}$, and the probability to observe a transition prior to τ_{max}. We denote these quantities as $\langle \tau | \tau < \tau_{max} \rangle$ and $P(\tau < \tau_{max})$, respectively. The true, unknown MFPT, $\langle \tau \rangle$, may be written as:

$$\langle \tau \rangle = P(\tau < \tau_{max}) \langle \tau | \tau < \tau_{max} \rangle + (1 - P(\tau < \tau_{max})) \langle \tau | \tau \geq \tau_{max} \rangle. \tag{S1}$$

Thus, it remains to evaluate the MFPT of trajectories with $\tau \geq \tau_{max}$, denoted $\langle \tau | \tau \geq \tau_{max} \rangle$, to obtain an estimation for $\langle \tau \rangle$. To evaluate $\langle \tau | \tau \geq \tau_{max} \rangle$, we assume that the probability density function of the process decays exponentially for times longer

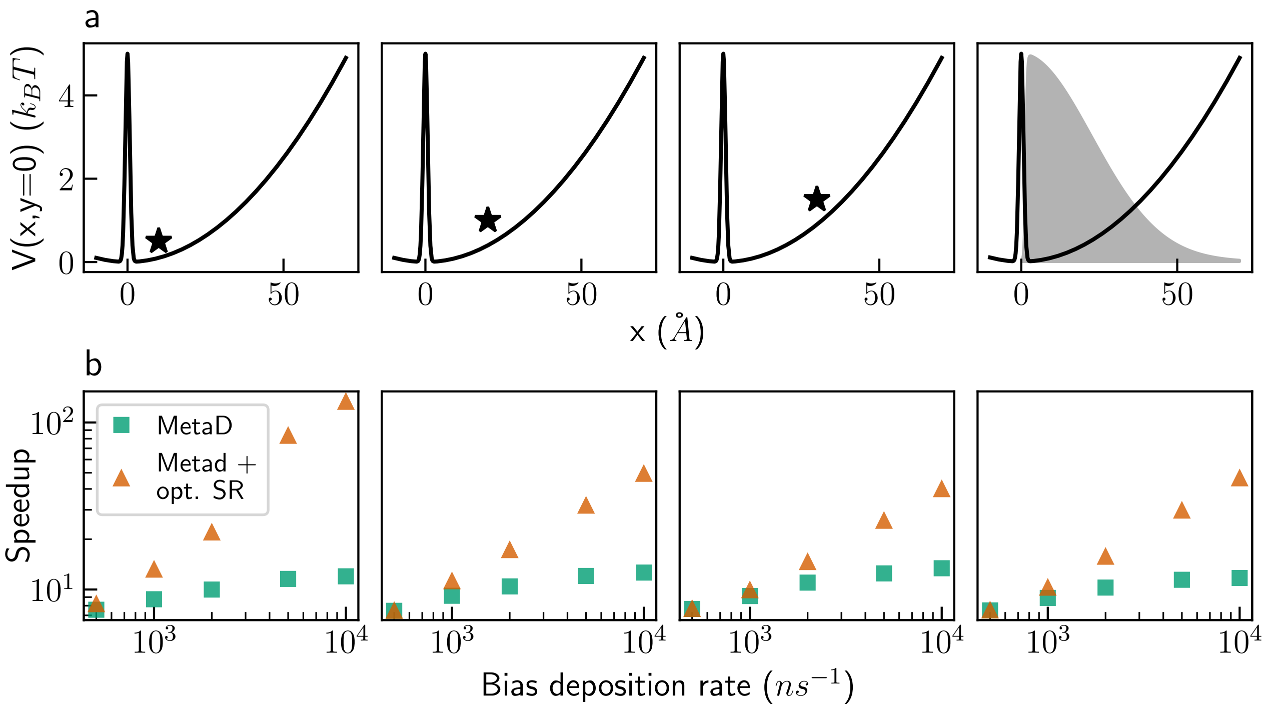

Supplementary Figure 1. Sensitivity to the initial positions. a Different initial positions along the x-axis on top of $V(x,y=0)$, with the potential $V(x,y)$ given in Equation 3 of the main text. The stars mark fixed initial positions and the shaded area marks the Boltzmann distribution, from which initial positions were sampled. **b** Speedup as a function of bias deposition rate for Metadynamics (MetaD) simulations without stochastic resetting (SR) and with optimal (opt.) SR, in green squares and orange triangles, respectively. Results are shown for different sets, from left to right: initiating at $(x=10,20,30,y=0)$ Å, and initiating at positions sampled from the Boltzmann distribution within the right well. Source data are provided as a Source Data file.

than some characteristic time τ_0. If $\tau_0 < \tau_{max}$, we can sample the rate of the decay from the tail of $P(\tau < \tau_{max})$. Practically, we plot the survival probability at times $\tau < \tau_{max}$ on a logarithmic scale and fit a linear function close to $\tau = \tau_{max}$. The slope of the fit is taken as the exponential rate μ, and is used to evaluate:

$$\langle \tau | \tau \geq \tau_{max} \rangle = \tau_{max} + \mu^{-1}. \quad \text{(S2)}$$

Substituting in Equation. S1 yields the estimated MFPT.

About 5% of MetaD simulations using the ϕ_2 angle as collective variable (CV) did not show a transition. The MFPT for this case was estimated as explained above for the unbiased case. For this angle, we also estimated the coefficient of variation (COV), which required the standard deviation. To obtain it, for each trajectory j that did not show a transition, we sampled a value η_j from an exponential distribution with rate μ and acquired a transition time $\tau_j = \tau_{max} + \eta_j$.

3 Transitions between sub-states of alanine tetrapeptide

Alanine tetrapeptide has 8 metastable states, defined using the ϕ_1, ϕ_2 and ϕ_3 dihedral angles[1,2]. Below, we adopt the labeling $S_1 \dots S_8$ given by Tsai et al.[2] In the main text, we follow transitions between two basins, separated along ϕ_3, which is the slowest degree of freedom. Simulations were initiated from the most stable state, S_8, and stopped when observing a transition into $0.5 < \phi_3 < 1.5$ rad, which corresponds to reaching either S_1, S_2, S_3 or S_4.

Here, to show that resetting can be useful also to transitions between multiple states in a multidimensional system, we explore transitions from S_8 to additional states: S_5, separated from S_8 by ϕ_2 only, S_4, separated by ϕ_3 only, and S_1, separated by both ϕ_2 and ϕ_3. Supplementary Fig. 2a shows the free-energy surface along ϕ_2 and ϕ_3 in the region $-3.14 < \phi_1 < -0.5$ rad,

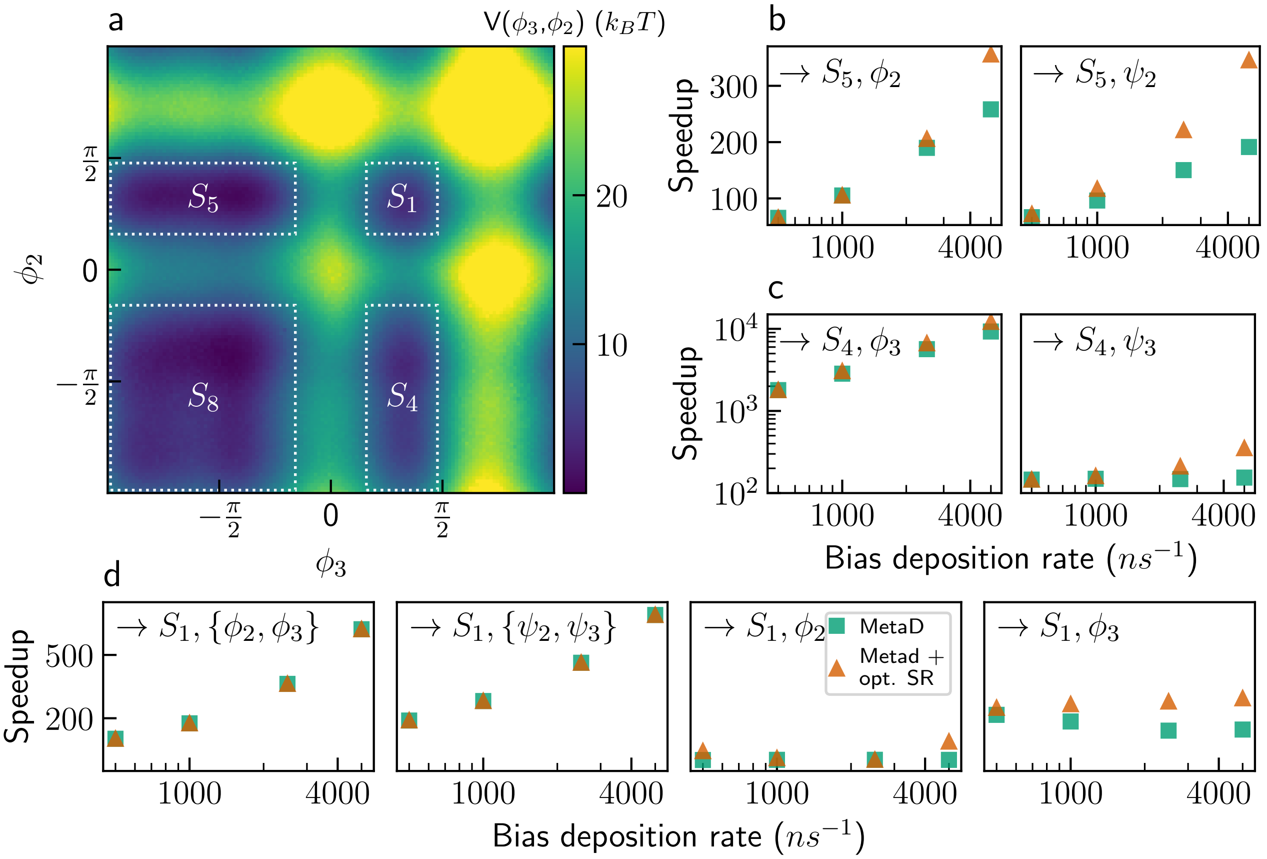

Supplementary Figure 2. Transitions between sub-states of alanine tetrapeptide. a Free-energy of alanine tetrapeptide along the ϕ_2 and ϕ_3 dihetral angles in the region $-3.14 < \phi_1 < -0.5$ rad. Dotted rectangles mark four stable states: S_1, S_4, S_5 and S_8. **b** Speedup as a function of bias deposition rate for transitions from S_8 to S_5, for Metadynamics (MetaD) simulations with no stochastic resetting (SR) (green squares) and with optimal (opt.) SR (orange triangles), using either ϕ_2 or ψ_2 as collective variable (CV). **c** Speedup as a function of bias deposition rate for transitions from S_8 to S_4, for MetaD simulations with and without SR, with either ϕ_3 or ψ_3 as CV. **d** Speedup as a function of bias deposition rate for transitions from S_8 to S_4, for MetaD simulations with and without SR, and different CVs: Both ϕ_2 and ϕ_3, both ψ_2 and ψ_3, ϕ_2 only and ϕ_3 only. Source data are provided as a Source Data file.

with dotted rectangles defining the relevant states. We performed MetaD simulations with different bias deposition rates and CVs. Angles ϕ_2/ϕ_3 and ψ_2/ψ_3 serve as good and suboptimal CVs, respectively, for transitions to S_5/S_4. We bias both $\{\phi_2, \phi_3\}$ or $\{\psi_2, \psi_3\}$ at the same time to obtain fast transitions to S_1. Simulations using ϕ_2 or ϕ_3 only serve as examples for the performance of MetaD when important degrees of freedom are not included. We note that these final sets of simulations, as well as unbiased simulations to determine the true MFPT values, include 10^3 trajectories, as opposed to 10^4 trajectories for all other sets of alanine tetrapeptide simulations. Some of the sets of 10^3 did not show a first-passage even after $10\,\mu s$. In these cases, we evaluated the MFPT and COV values by the procedure detailed in Section 2.

Supplementary Figures 2b, 2c and 2d show the speedup obtained for transitions to S_5, S_4 and S_1 respectively, with MetaD and no SR (green squares) and with optimal SR (orange triangles). We observe the same phenomena described in the main text, mainly, SR provides further accelerations for suboptimal CVs. It also accelerates some simulations with good CVs, especially when high bias deposition rates are used.

4 Kinetics inference for infrequent MetaD simulations with SR

The kinetics inference procedure for infrequent MetaD (iMetaD) simulations with SR is composed of the following steps: First, we obtain a set of N trajectories ending in a first-passage from iMetaD simulations with SR. We divide the full trajectories to

the shorter trajectories between resetting events. These strips form a set of $M_{tot} \geq N$ shorter iMetaD trajectories, N of them successfully ending with a first-passage. The length of each strip $t_j = n_j \Delta t$ is scaled using the standard iMetaD acceleration factor α_j,[3,4] to $\tilde{t}_j = \alpha_j t_j$, where

$$\alpha_j = \frac{1}{n_j} \sum_{i=1}^{n_j} e^{\beta V_j(s(t_i),t_i)}. \tag{S3}$$

Here, n_j is the total number of time steps in the strip, Δt is the time step size, V_j is the external bias potential, s is the CV, t_i is the i-th time step, and β is the inverse temperature.

Next, we evaluate the survival function, defined as $S(\tilde{t}) = M_{\{\tilde{t}_j > \tilde{t}\}}/M_{tot}$ with $M_{\{\tilde{t}_j > \tilde{t}\}}$ being the number of strips with rescaled length that is larger than $\tilde{t}$. Finally, we assume that the underlying true first-passage time (FPT) distribution is exponential. For an exponential FPT distribution, $\log(S(\tilde{t}))$ decays linearly with a slope of $-\langle\tau\rangle^{-1}$, where $\langle\tau\rangle$ is the MFPT. We perform a linear fit to the obtained survival function, and use its slope to estimate the unbiased MFPT. To include all first-passage transitions in the analysis, we took the maximal $\tilde{t}$ to be smaller than the $\tilde{t}_j$ of the shortest trajectory that did not show a transition.

This procedure is demonstrated for alanine tetrapeptide. Supplementary Fig. 3a shows $\log(S(\tilde{t}))$ for iMetaD simulations and no SR using the sub-optimal CV ψ_3 (in green). The true survival function, as obtained from unbiased simulations, is given in blue. Due to bias over-deposition, the resulting survival function decays much slower than the true one. However, at short times, where the bias is minimal, even a sub-optimal CV gives a survival function that tracks the unbiased one. The benefit of resetting is in providing excessive sampling of the short time region, leading to more reliable estimation of the exponential decay of the survival. The survival function estimated from iMetaD with SR is given in Supplementary Fig. 3b, showing an improved agreement with the unbiased results. The quality of the linear fit (black line) provides an assessment for the reliability of the results. Specifically, we use the Pearson correlation coefficient R between the samples and the linear fit. Supplementary Fig. 4 shows R^2 next to the associated prediction error, as a function of resetting rate. It reaches $R^2 \to 1$ for high resetting rates, leading to minimal error.

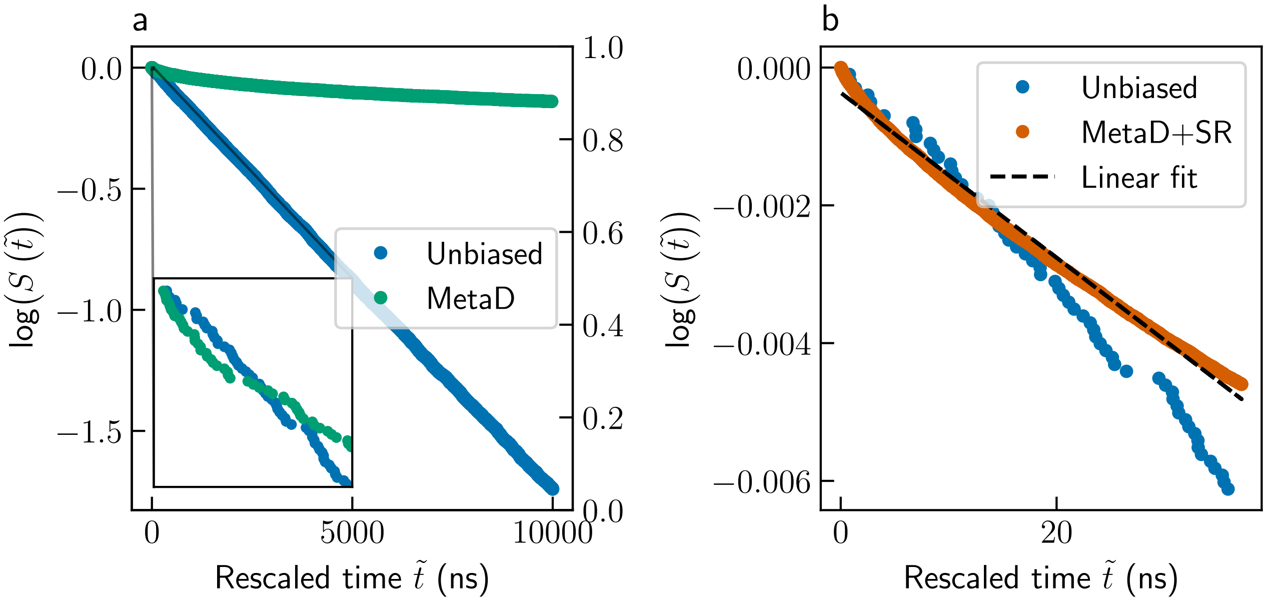

Supplementary Figure 3. Survival functions of Metadynamics trajectories with resetting. a The survival function for unbiased trajectories (blue) and Metadynamics (MetaD) trajectories (green). The inset shows a zoom in on short times. **b** The survival function for MetaD trajectories with stochastic resetting (SR) (orange). A linear fit is shown in black. Source data are provided as a Source Data file.

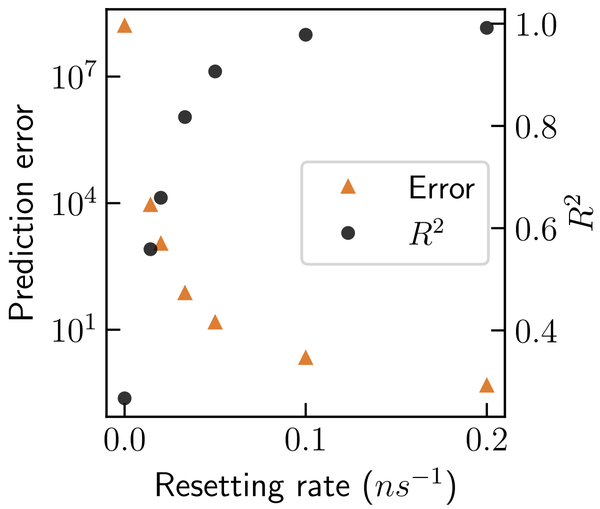

Supplementary Figure 4. Reliability assessment. Prediction error (orange triangles) and R^2 values (black circles) as a function of resetting rate. Source data are provided as a Source Data file.

Supplementary References

1. Invernizzi, M. & Parrinello, M. Exploration vs convergence speed in adaptive-bias enhanced sampling. *J. Chem. Theory Comput.* **18**, 3988–3996, DOI: 10.1021/acs.jctc.2c00152 (2022).

2. Tsai, S.-T., Smith, Z. & Tiwary, P. Sgoop-d: Estimating kinetic distances and reaction coordinate dimensionality for rare event systems from biased/unbiased simulations. *J. Chem. Theory Comput.* **17**, 6757–6765, DOI: 10.1021/acs.jctc.1c00431 (2021). PMID: 34662516, https://doi.org/10.1021/acs.jctc.1c00431.

3. Tiwary, P. & Parrinello, M. From metadynamics to dynamics. *Phys. Rev. Lett.* **111**, 230602, DOI: 10.1103/PhysRevLett.111.230602 (2013).

4. Valsson, O., Tiwary, P. & Parrinello, M. Enhancing important fluctuations: rare events and Metadynamics from a conceptual viewpoint. *Annu. Rev. Phys. Chem.* **67**, 159–184, DOI: 10.1146/annurev-physchem-040215-112229 (2016).
